# Impact of the mixed degree of urban functions on the taxi travel demand

**DOI:** 10.1371/journal.pone.0247431

**Published:** 2021-03-04

**Authors:** Changwei Yuan, Yaxin Duan, Xinhua Mao, Ningyuan Ma, Jiannan Zhao

**Affiliations:** 1 College of Transportation Engineering, Chang’an University, Xi’an, China; 2 Engineering Research Center of Highway Infrastructure Digitalization, Ministry of Education, Xi’an, China; 3 Xi’an Key Laboratory of Digitalization of Transportation Infrastructure Construction and Management, Xi’an, China; 4 Department of Civil and Environmental Engineering, University of Waterloo, Waterloo, Canada; Institute for Advanced Sustainability Studies, GERMANY

## Abstract

As an important service industry in cities, taxis provide people with an all-weather travel mode. And its demand is greatly affected by the internal functions of the city. It is very important to understand the relationship between the mixed degree of urban internal functions and the residents’ taxi travel demand to alleviate traffic congestion and formulate corresponding urban traffic strategies. This paper combined two heterogeneous data in the main urban area of Xi’an, urban points of interest (POIs) and taxi GPS. Firstly, a spatial information entropy model was constructed to quantitatively evaluate the mixed degree of functions in different spaces within the city. Secondly, the kernel density estimation method was used to analyze the spatial distribution evolution characteristics of residents’ taxi travel demand. A geographically weighted regression (GWR) model was further used to study the spatial and temporal influences of the mixed degree of urban internal functions on taxi travel demand. Results indicate that there is an obvious spatiotemporal pattern in the impact of the mixed degree of urban functions on taxi travel demand. And the GWR model is used to study the impact is superior to the ordinary least squares (OLS). In more developed areas, improving the mixed degree of urban functions will be more attractive than backward areas. It is also found that although the single function of the city has an impact on the taxi travel demand, the result of the single function is not ideal. This study can provide a reference for the optimal combination of basic units of urban space in urban planning, promote the balance of supply and demand of urban taxis, rationalize urban taxis’ operation and allocation, and solve the problems of urban transportation systems.

## 1. Introduction

Taxis, as an important part of urban transport with flexible and convenient conditions, provide all-weather travel services, and play an indispensable role in urban resident trips [1, 2]. In recent years, with the expansion of the urban population and diversification of social activities, people’s demand for taxi travel has increased dramatically. In addition, with the gradual standardization of online taxi booking, taxi passenger volume has grown substantially. According to the 2018 Xi’an transport development annual report, there were 12,535 taxis in the city, carrying 361 million passengers in 2018, and an average of 990,000 passengers per day in 2018 [3]. However, due to the imbalance of the spatial distribution of pedestrian flow and the uncertainty of travel time, the taxi travel demand has a complex space-time dependence. Additionally, if taxi drivers do not have full knowledge of high-demand areas, it will inevitably lead to a significant imbalance between supply and demand in the taxi industry, traffic congestion, long waiting times of passengers, and other such phenomena. Therefore, it is necessary to dig deep into the residents’ taxi travel demand.

However, exploring the taxi travel demand has always been the focus of academic circles. Firstly, considering that residents’ taxi travel demand has obvious temporal and spatial distribution characteristics, how to use taxi GPS data to clarify these temporal and spatial distribution characteristics is an important basis. Secondly, the purpose of residents’ taxi travel is diverse, which is largely affected by the interaction of urban internal functions. In order to measure the interaction of this kind of urban internal functions, we introduce the concept of the mixed degree of urban functions. It is composed of urban points of interest (POIs), the basic units of urban space. And in recent years, web crawler technology has been widely used in various fields [4–6], providing important technical support for obtaining the data related to these urban POIs. These open urban POIs data can be used as the important data source for studying the urban internal functions [7, 8]. Then, how to use the urban POI data to quantitatively evaluate the mixed degree of urban functions and study its impact on the temporal and spatial dynamic changes of taxi travel demand is still lacking.

Therefore, this paper uses urban POI data and taxi GPS data to explore the relationship between the mixed degree of urban functions and residents’ taxi travel demand. Based on GIS technology, a spatial information entropy model is constructed to measure the mixed degree of urban functions and realize the spatialization of the information entropy model. The paper also uses Python big data analysis technology and kernel density estimation method to analyze the spatial distribution and evolution characteristics of taxi travel demand. Finally, the geographically weighted regression (GWR) model is used to verify the spatial-temporal differentiation pattern of residents’ taxi travel behavior and the mixed degree of urban functions. This study can offer a reference for the optimal combination of basic units of urban space in urban planning. Our work also provides a decision-making basis for promoting the balance of urban taxi supply and demand, solving the development of the urban taxi industry, and alleviating the urban traffic system problems in the formulation of traffic strategy.

The remainder of this paper is as follows. Section 2 summarizes the spatial-temporal variation characteristics of residents’ taxi travel, the influencing factors, and research methods. Section 3 outlines the research methods used in this article, including the spatial information entropy for the mixed degree of urban functions measurement, the kernel density estimation for taxi travel demand measurement, and the GWR model. Section 4 introduces the study area and data, including urban POI data and taxi GPS track data. Section 5 provides the model result and discussion. Section 6 summarizes the conclusions. Section 7 provides the limitations and future work.

## 2. Literature review

In recent years, with the popularization of mobile application technology and taxi GPS devices, a large amount of data related to individuals’ positions and trajectories has spring up [9]. All these provide an important scientific basis for the refined study of the spatial and temporal distribution characteristics of residents’ taxi travel. For example, in order to explore the mode of travel demand in New York City and improve the efficiency of taxi companies, Tang et al. [10] analyzed the spatial and temporal distribution characteristics of travel demand in different regions by extracting the location data of passenger points from the GPS track data of New York City taxis. Based on the hidden Markov model, Alvarez-Garcia et al. [11] analyzed the temporal and spatial characteristics of taxi driving using past GPS logs and current location. Bischoff et al. [12] analyzed the travel behavior and vehicle supply of the Berlin taxi market based on the GPS data of taxis. And the time analysis showed that there would be peak demand in the morning and afternoon of weekdays, and the peak demand would turn to night on weekends. The spatial analysis showed that most taxi travels involved city centers and airports. All of these studies conducted beneficial exploration of the spatiotemporal variation rules and behavior patterns of residents’ taxi travels.

On this basis, many scholars further discussed the influencing factors of the spatial and temporal distribution characteristics, which were mainly divided into three categories. The first category is the urban built environment factors. For example, Zhang et al. [13] took urban built environment, road length, road density, residential building quantity, residential building density, employment place, and public service as important factors to analyze the temporal and spatial impact of taxi traffic by using the geographically and temporally weighted regression (GTWR) model. Wu et al. [14] studied the spatial distribution characteristics of taxi routes in downtown Shanghai and established a multiple linear regression model to quantitatively analyze the impact of the urban construction environment on short-distance taxi trips in the city. The second category is the weather factors. For example, Kamga et al. [15] analyzed the impact of weather changes on the balance of supply and demand of taxis based on a 10-month GPS data set of a taxi in New York City, and found that rainy weather often leads to higher passenger capacity. Based on the data of taxis, meteorology, and air quality, Kang et al. [16] conducted a spatial-temporal analysis of residents’ taxi travel demand and loading and unloading areas in different weather and revealed the relationship between the weather and the taxi travel activities of residents. The third category is the land-use factors. Jiang et al. [17] established a linear relationship between taxi travel behavior and land-use using taxi track data and POI data of urban interest points. Liu et al. [18] used taxi track data to study the variation of passenger volume over time and the relationship between this and different land-use characteristics. The research results are helpful for urban planners and decision-makers to alleviate the issues of transportation and resources.

In the analysis of the influencing factors of residents’ taxi travel demand, the most traditional methods are the ordinary least squares (OLS) multiple regression model [19–21] and the four-stage method [22]. An important assumption of the ordinary least squares multiple regression model is that all variables in the study area are stable and independent, and the variance of the error term is the same [23]. The method is characterized by fast and convenient calculation, which is more suitable for analyzing the travel demand of specific travel modes. However, due to the different purposes of residents’ taxi travel and the spatial differences of urban internal functions, local spatial changes will not be captured and the accuracy of model results will be reduced if such spatial differences are ignored. In order to overcome this defect, some scholars have utilized the GWR model. Since the GWR model explains the spatial distribution differences of the research objects well [24–26], it has been widely used in the analysis of spatial heterogeneity [27–30].

To sum up, the aforementioned studies mainly used GPS track data of taxis to analyze the spatial and temporal distribution characteristics of residents’ taxi travel and the influencing factors of such characteristics. These studies set each factor as a single variable, where influencing factors’ combined effects are not comprehensively considered. However, the taxi travel demand of urban residents, that is, the purpose of travel, is largely affected by the interaction of urban internal functions. Due to their continuous development, it is hard to divide a city into a set of single functional areas, which can easily cause the disconnection of various elements of urban ecology. We should recognize the connection and importance of multi-functional living environments and social interaction spaces [31]. In this situation, function mixing has appeared in cities. Urban function mixing is to compact arrange a series of interrelated functions in the same area, so as to reduce the travel cost and effectively improve the efficiency and public welfare level of the city [32]. Therefore, how to measure the mixed degree of urban functions and its influence on residents’ taxi travel demand is a problem worth discussing.

## 3. Methods

**Fig 1:**
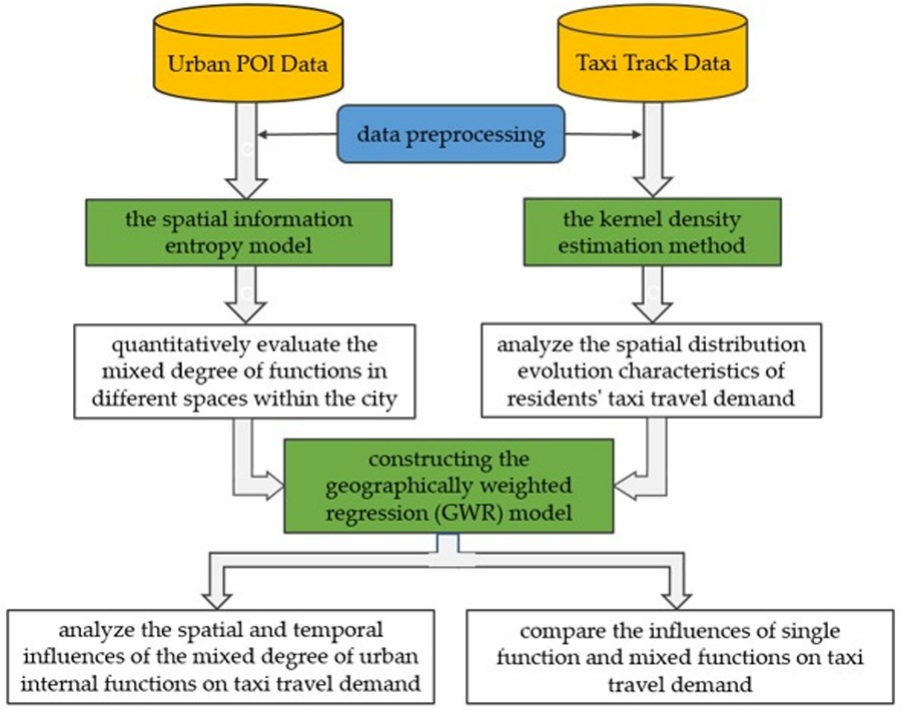
Research framework.

By presenting the concept of the urban function mixing, we use the ArcGIS and Python software to build a spatial information entropy model to measure the mixed degree of urban functions. We further use the kernel density estimation method to analyze the spatial distribution and evolution characteristics of taxi travel demand during the three peak periods of the morning, afternoon and evening on weekdays and weekends. Then, through the GWR Model, the spatial and temporal differences of the impact of the mixed degree of urban functions on taxi travel demand are further discussed by using regression coefficient. Finally, the influences of single function and mixed functions on taxi travel demand are compared. The overall research framework of the paper is shown in Fig 1.

### 3.1 Spatial information entropy for the mixed degree of urban functions measurement

The information entropy model is one of the most commonly used diversity measurement methods in the evaluation of mixed-use [33, 34]. Generally, the larger the information entropy of a system, the stronger the mixing of the system. Conversely, the smaller the information entropy, the weaker the mixing property of the system. Therefore, information entropy can be used to describe the mixing degree of a system. The formula of information entropy is

$$H=-\sum_{i=1}^{n}p_{i}\;log\;p_{i}$$ (1)

where *p*_*i*_(1,2,3,‥,*n*) is the probability that a system is in different states, 0 ≤ *p*_*i*_ ≤ 1.*n* stands for different states.

Since a city contains a significant amount of information, it can be regarded as a spatial system where the urban POIs are denoted as various functional attributes. Then, we introduce the concept of information entropy at the spatial level, construct the spatial information entropy model, and use the information entropy to measure the mixed degree of different spatial functions in the city. The steps are as follows.

**Fig 2:**
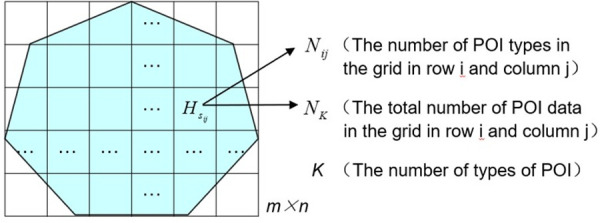
Grid treatment of urban space.

**Step 1:** The urban space is gridded by ArcGIS software. A rectangle that can cover the entire urbanized area is designed and then divided into m × n non-intersecting squares of equal size of a m × a m (as shown in Fig 2, the blue part is assumed to be the urbanized area to be studied). The analysis unit does not adopt the traditional traffic analysis area, such as administrative division [35], block [36], or community [37], for three main reasons. The first reason is that the traditional traffic area is too large and the scale unit is not sufficiently fine. The second reason is that a grid allows the use of units that can be controlled, thus providing a reasonable scale to describe and understand the spatial distribution of the research objects. The third reason is that the grid processing is convenient for computing and allows the processing of a large amount of spatial data quickly.

**Step 2:** Constructing the information entropy model of urban functional space. Let the total type number of POI is *K*. In the grid of row *i* and column *j*, the number of certain POI types is *N*_*ij*_, and the total number of POIs in this grid is *N*_*K*_. Then in the grid of row *i* and column *j*, the ratio of the number of certain POI types in the total number is *p*_*ij*_ = *N*_*ij*_/*N*_*K*_, *Σ*_*i*_*Σ*_*j*_*p*_*ij*_ = 1. Therefore, the information entropy model of the constructed urban functional space is

$$H_{s_{ij}}=-\sum_{i}^{m}\sum_{j}^{n}p_{ij}\times log\;p_{ij}\;i=1,2,3,\ldots ,m\;j=1,2,3,\ldots ,n$$ (2)

where *H*_*s*_ is the spatial information entropy, and *H*_*s*_ ≥ 0, because 0 ≤ *p*_*ij*_ ≤ 1. The higher the value of *H*_*s*_, the greater the difference in function types within the city, and the smaller the difference in the number of each function type.

**Step 3:** Calculation of the mixed degree of urban functions. Through ArcGIS software, we can traverse the number of different types of POI data in each grid cell to obtain the values of each *p*_*ij*_ and each *H*_*s*_. Namely, the functional mixing degrees of cities in different spaces have been obtained.

### 3.2 Kernel density estimation for taxi travel demand measurement

#### 3.2.1 Taxi travel demand measurement method

**Fig 3:**
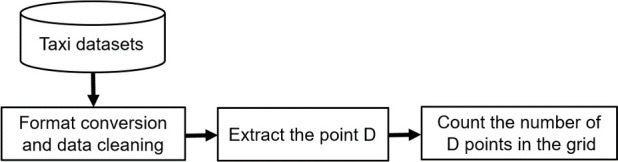
The calculation process of taxi travel demand.

Fig 3 presents the flow chart of the measurement method of taxi travel demand in this paper. First of all, it is necessary to transform and preprocess the acquired GPS track data file. Format conversion is used to convert the acquired original data file type into a file type that can be used to carry out data analysis. Preprocessing includes correction and elimination of duplicates, invalid data, data outside the research scope, and incomplete information.

Then, we extract the OD (Here, point O, where the vehicle operation status changes from "empty vehicle" to "full vehicle". Point D, where the vehicle operation status changes from "full vehicle" to "empty vehicle".) points of the cleaned taxi data. Here, we only extract the data relating to the destination (point D), to represent a residents’ travel purpose and hence taxi demand.

Finally, coordinate transformation and map matching are carried out, and the number of points D in each grid cell that meshes the urban space is counted respectively.

#### 3.2.2 Kernel density estimation method

**Fig 4:**
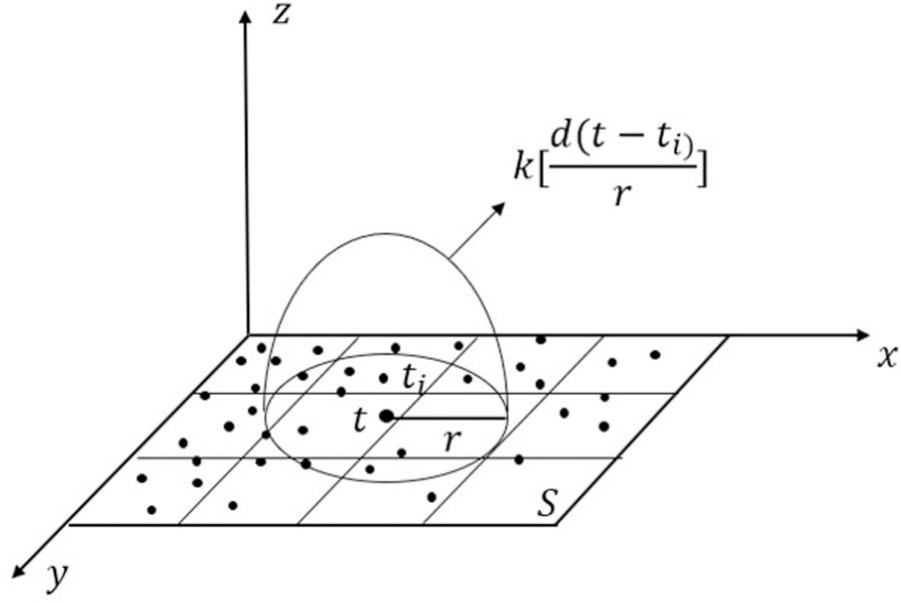
Spatial kernel density estimation.

In order to reflect the spatial distribution and evolution characteristics of residents’ taxi travel demand more directly, this paper adopts the kernel density estimation method to analyze the variation of taxi travel demand density. The kernel density estimation method can be used to mine spatial data. It cannot only select the appropriate bandwidth flexibly according to the research scale but also has certain advantages in the aspect of detailed local characteristics. Using the first law of geography, this method holds that the closer the distance between things, the greater the density expansion value obtained from the proximity to the core elements, thus fully reflecting the spatial distribution position difference of the research object [38]. Accordingly, the kernel density estimation method is used to characterize the spatial distribution characteristics and rules of taxi boarding and alighting points. The spatial kernel density estimation is provided in Fig 4.

The calculation method of kernel density estimation is as follows:

$$f\left(t\right)=\frac{1}{nr^{2}}\sum_{i=1}^{n}k\left[\frac{d\left(t-t_{i}\right)}{r}\right]$$ (3)

where *f*(*t*) is the kernel density estimation function at the spatial position point *t*. *r* is the search bandwidth, that is, the search radius, which is determined according to the dispersion degree of taxi point D and the analysis scale. *t*_*i*_(*i* = 1,2,3,…*n*) represents all observation points. *n* is the number of all observation points whose distance from the point *t* ≤ *r*. $k\left[\frac{d\left(t-t_{i}\right)}{r}\right]$ is the kernel function of spatial weight. *d*(*t*–*t*_*i*_) is the distance between point *t*_*i*_ and point *t*. The larger the value of *d*(*t*–*t*_*i*_), the smaller the spatial weight kernel function.

In this paper, the most widely used Gaussian kernel function is used to determine the spatial weight. The calculation method is as follows:

$$k\left[\frac{d\left(t-t_{i}\right)}{r}\right]=\frac{1}{\sqrt{2\pi }}exp\left[-\frac{d^{2}\left(t-t_{i}\right)}{2r^{2}}\right]$$ (4)

### 3.3 Geographically Weighted Regression (GWR) model

The geographically weighted regression (GWR) model is an extension of the general linear regression model. It incorporates the spatial location of data into the regression model, which can reflect the driving influence change of the research object in the spatial dimension [25]. In this paper, the geographically weighted regression (GWR) model is used to analyze the impact of the mixed degree of urban functions on the taxi travel demand of residents. The number of taxi travel demand D points is taken as the dependent variable. And the measurement value of the mixed degree of urban internal functions is taken as the explanatory variable. The calculation formula of the model is as follows:

$$y_{i}=\beta_{0}\left(u_{i},v_{i}\right)+\sum_{i=1}^{n}\beta_{i}\left(u_{i},v_{i}\right)x_{i}+\varepsilon_{i}\;i=1,2,3,\ldots ,n$$ (5)

where *i* is the cross section. *y*_*i*_ is the number of residents’ taxi travel demands in the *ith* grid cell. (*u*_*i*_, *v*_*i*_) represents the geographical coordinates of the geometric center of the *ith* grid cell. *β*_0_(*u*_*i*_, *v*_*i*_) is the regression constant of the *ith* grid cell. *β*_*i*_(*u*_*i*_,*v*_*i*_) is the regression parameter of the *ith* grid cell. *n* is the total number of grid cells, that is, the total measure of the mixed degree functions. *x*_*i*_ is the value of the mixed degree functions in the *ith* grid cell. *ε*_*i*_ is the residual term of the model, with a mean value of zero and variance of *δ*^2^.

## 4. Study area and data

### 4.1 Study area

**Fig 5:**
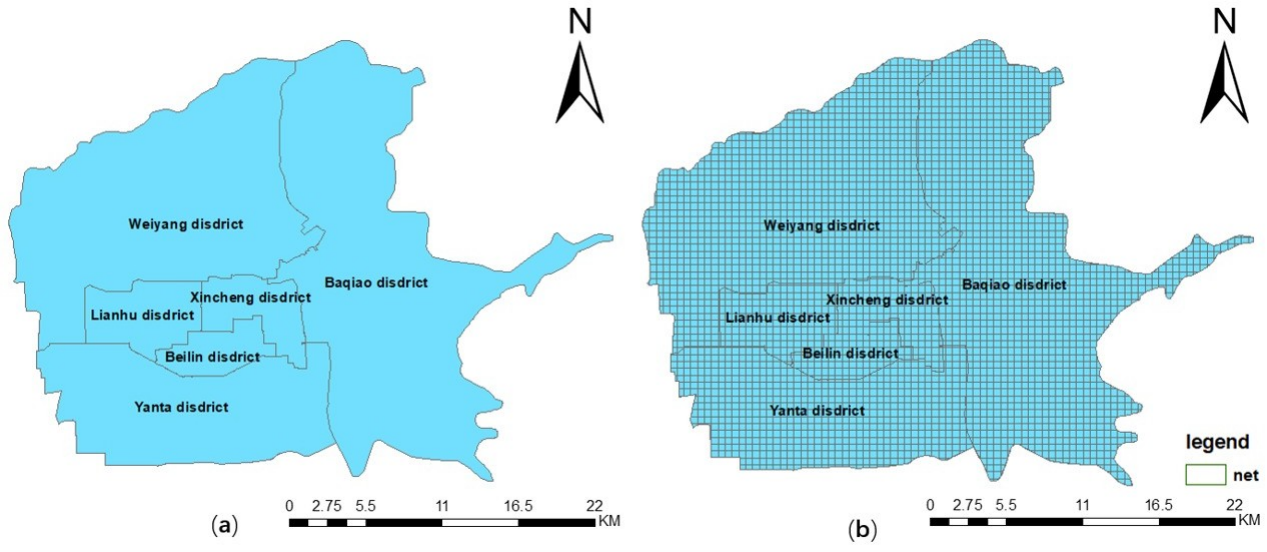
Study area. (a) Administrative divisions in the study area; (b) grid generation of the study area.

Xi’an is an important central city in the western region of China. By the end of 2018, Xi’an had a total area of 10,096.81 square kilometers, a permanent resident population of 10.037 million, and a well-developed transportation network. According to the 2018 traffic analysis reports for major cities in China, Xi’an had the highest proportion of public trips in the country in 2018 [39]. As an important part of public transport travel in Xi’an, the main urban area of Xi’an has a relatively large demand for taxi travel, representing 90% of the demand for taxi travel in the wider city. Therefore, this study took the main urban area of Xi’an as the study area, including the districts of Weiyang, Lianhu, Xincheng, Beilin, Yanta, and Baqiao (Fig 5A). In order to study the influence of the mixed degree of urban internal functions on taxi travel demand, after many attempts, the main urban area of Xi’an was divided into a grid of 500 × 500 m squares as the analysis unit. One is that it is more accurate in spatial structure study. Secondly, when a city is taken as the research area to study the taxi travel demand problem, it is usually divided into 500 m×500 m square grids [13, 29, 40]. The total number of analysis units was 3486 (Fig 5B), which is similar to the scale of the street units used in planning research.

### 4.2 Data sources

The data sources used in this paper include the urban POI data collected from the Gaode Map and the GPS track data of taxis obtained from the taxi management office of Xi’an city.

#### 4.2.1 Urban POI data

The urban POIs describes the spatial and attribute information of geographical entities [41]. It is a basic unit that reflects various functions within a city, the spatial distribution of urban functions, and residents’ travel purposes [42, 43]. The urban POI data used in this paper were from the Xi’an Gaode Map of 2019, and the POI data was collected using the Gaode Map. After coordinate transformation and address matching of the original data, a total of 308,450 POIs were obtained in the main urban area of Xi’an city. Each POI data point was captured with ten attributes: id, name, telephone, address, longitude coordinates, latitude coordinates, type, province, city and district. Here we only show 10 sets of data (Table 1). And telephone numbers are no longer displayed because of privacy issues.

**Table 1:** POI data structure.

ID	Name	Telephone	Address	Longitude coordinates	Latitude coordinates	Type	Province	City	District
B001D06F4Y	Fujian Tanyang Kungfu Tea Factory	********	No.2, Donglin street, Xinhua wholesale market, Tumen Grove	108.881516	34.264303	Specialty stores	Shaanxi	Xi’an	Lianhu
B0FFFFUKWF	Agent of Xupai Battery in West District	********	150m south of the intersection of Tuanjie middle road and Fenghui Road	108.889257	34.263164	specialty stores	Shaanxi	Xi’an	Lianhu
B0FFFZ6BM4	Dongfang Motor Company	********	Tuanjie Middle Road, opposite to China Telecom	108.888419	34.263999	specialty stores	Shaanxi	Xi’an	Lianhu
B0FFH332EE	Shida Tool	********	No. A10 of Yuanfeng electronic market	108.890128	34.266521	specialty stores	Shaanxi	Xi’an	Lianhu
B001D09N54	Wave Glasses	********	North of 1st floor, no.6-7, No.42, FengHao East Road	108.890494	34.258641	specialty stores	Shaanxi	Xi’an	Lianhu
B0FFGF7JSB	Herunming Tea	********	50 meters west at the intersection of Fengdeng South Road and Fengdeng West Road	108.894368	34.256686	specialty stores	Shaanxi	Xi’an	Lianhu
B0FFHLWR3J	New Century Music Bookstore	********	No.8, Row 6, 2nd floor, Baihui market, Chang’an Middle Road	108.9462	34.22756	specialty stores	Shaanxi	Xi’an	Yanta
B0FFHRPLHG	Jinzang Xifeng Wine & Hengtai Tobacco and Wine	********	Huacheng International, No.82, Chang’an West Road, electronic city street	108.94463	34.191275	specialty stores	Shaanxi	Xi’an	Yanta
B0FFHUGV90	Denghua Tobacco and Wine Convenience Store	********	No. 82, Chang’an South Road, Huacheng International Community, 7–20109	108.94564	34.192836	specialty stores	Shaanxi	Xi’an	Yanta
B0FFKS02J4	Jindingsheng tobacco and Wine	********	East side of the north gate of ivory Palace on Tiantan West Road	108.945773	34.201899	specialty stores	Shaanxi	Xi’an	Yanta

**Fig 6:**
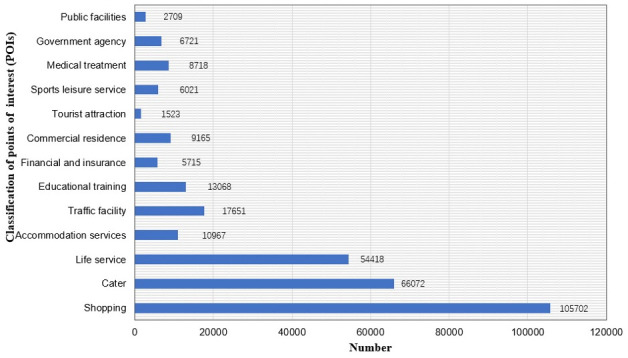
Data distribution of urban points of interest (POIs).

According to the basic attributes of POI data, urban functional areas were divided into 13 categories: cater POI, tourist attraction POI, public facility POI, shopping POI, traffic facility POI, educational training POI, financial and insurance POI, commercial Residence POI, life service POI, sports leisure service POI, medical treatment POI, government agency POI, accommodation services POI (Table 2 and Fig 6).

**Table 2:** Classification of points of interest (POIs) in this study.

Number	Classify	Details
1	Cater	Chinese restaurant, Western restaurant, snack fast food, cake dessert shop, drink shop, coffee shop, etc.
2	Tourist attraction	park, square, botanical garden, zoo, temple, scenic spot, etc.
3	Public facility	public toilets, kiosks, public telephone, emergency shelters, etc.
4	Shopping	shopping malls, shopping centers, supermarkets, home appliance and electronics stores, home furnishing materials, specialty stores, etc.
5	Traffic facility	railway station, high-speed railway station, parking lot, bus station, subway station, bus station, etc.
6	Educational training	kindergartens, primary and secondary schools, colleges and universities, adult education, training institutions, etc.
7	Financial and insurance	insurance companies, finance companies, ATMs, securities compa nies, bank, etc.
8	Commercial Residence	residential quarters, commercial office buildings, dormitories, etc.
9	Life service	manicure and hairdressing, photo studio, public toilet, telecommunication business hall, laundry, maintenance point, etc.
10	Sports leisure service	sports venues, cinema, KTV, Internet bar, bath and massage, farmhouse, etc.
11	Medical treatment	general hospitals, specialized hospitals, clinics, pharmacies, experience institutions, animal medical facilities, etc.
12	Government agency	government agencies, public security organs, social organizations, traffic vehicle management, foreign institutions, etc.
13	Accommodation services	hotels, guest houses, youth hotels, etc.

#### 4.2.2 Taxi track data

Taxi track data can reflect urban traffic operation, residents’ activity rules, and urban functional spatial structure. Except for special events, the number of trips per week is stable, and there is a repeated pattern every week [27]. Therefore, this paper used more than 2.7 million taxi track data from September 4th to September 10th, 2019 in Xi’an city for analysis. No major holidays occurred during the analyzed period, thereby eliminating the impact of contingencies, single day analysis errors, and thus allowing a complete appreciation of the characteristics of residents’ taxi travel demand. The obtained driving track data of each taxi included vehicle ID, time, direction, longitude and latitude, effectiveness, state, and other information. The specific data format is presented in Table 3.

**Table 3:** Taxi track data structure.

Name	Sample	Meaning
Vehicle ID	AU3502	The issuance of vehicle license plates
Time	20191104082015	GPS_time
Longitude	108.923815	Location information
Latitude	34.340936	Location information
Direction	90	Front direction
EFF	1	Vehicle state bit; 1 means valid, 0 means invalid
State	4	Vehicle state; 4 means empty, 5 means someone in the vehicle

Since the selected study area was the main urban area of Xi’an, it was necessary to extract and preprocess the obtained taxi track data of Xi’an to ensure consistency between the study area and the data coverage. The specific steps were as follows:

- Eliminating duplicate, invalid and data outside the main urban area of Xi’an city.
- Removing the data of travel time t < 2 min or t > 220 min.
- Extracting the data of point D from the OD point, that is, the point at which the vehicle operation state changes from a full vehicle to an empty vehicle (i.e., from state 5 to state 4).

After the above steps, a total of 2.4 million effective taxi tracks were obtained within the main urban area of Xi’an for one week.

## 5. Results and discussion

### 5.1 Measurement results of the mixed degree of urban functions

**Fig 7:**
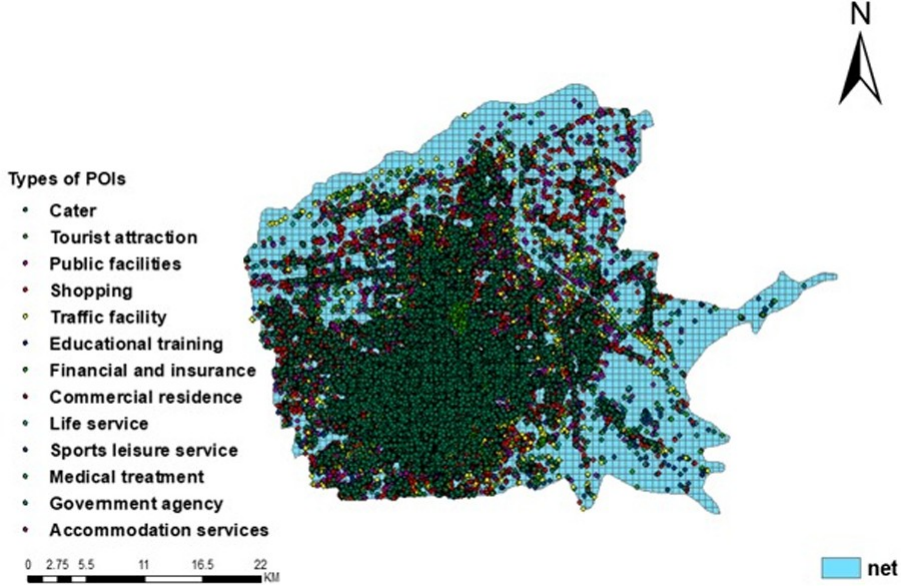
The spatial distribution of 13 categories of POI data.

In order to show the spatial distribution of POIs in Xi’an more clearly, GIS software was used to visualize the POI data divided into 13 categories (Fig 7).

**Fig 8:**
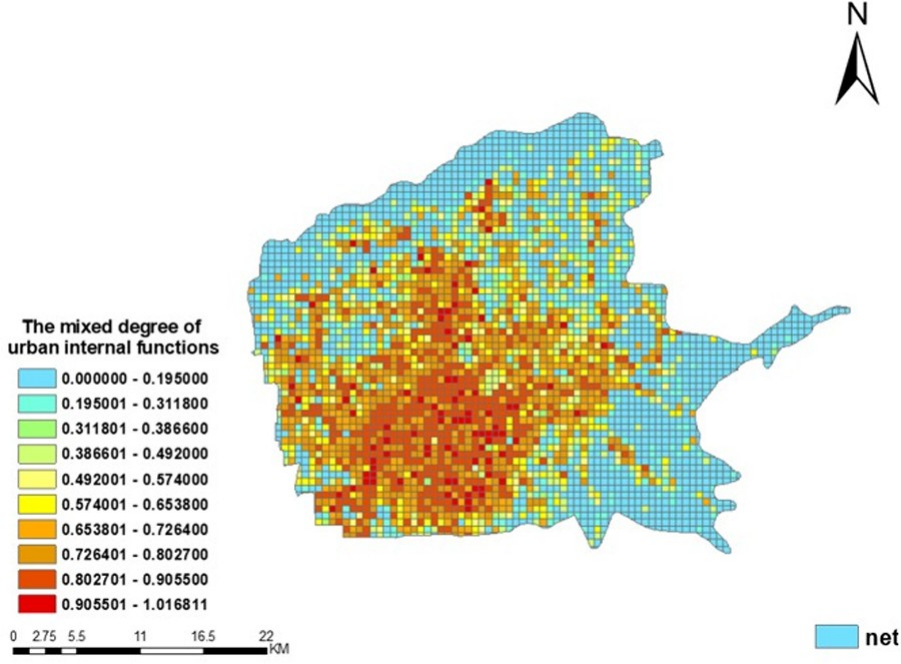
The mixed degree of functions in the main urban area of Xi’an.

Furthermore, according to the spatial information entropy model proposed above and the measurement method of the mixed degree of functions in different spaces within the city, this paper used the regional statistical tool in the ArcGIS toolbox to calculate the spatial distribution of the 13 categories of POI data, and obtained the mixed degree of urban functions in each grid cell (Fig 8). It can be seen that within the main urban area of Xi’an there is a certain spatial difference in the mixed degree of urban functions. And the multicenter characteristics are obvious. Overall, the mixed degree is lowest outside the third ring road and gives a trend of gradual increase within the third ring road. In particular, the mixed degree of urban POIs in some places outside the third ring road is as low as 0, but within the third ring road, the maximum is 1.0168.

Among the areas of focus, the traditional Bell Tower business circle in Beilin district, Xiaozhai business circle in Yanta district, Exhibition Center, and High-Tech Road have the highest mixed degree, up to 0.8028–1.0618. This is because these areas have a large number of shopping malls, entertainment facilities, office buildings, restaurants, etc., showing diversity. These regions also have a relatively high proportion of taxi demand. One possible explanation is that more mixed areas are more attractive than others. The mixed degree of urban POIs is the second-highest in the area around the newly rising North City business circle in Weiyang district. These also reach more than 0.6539. In addition, the mixed degree of urban POIs near Xi’an High-Speed Railway station is also relatively low, mostly below 0.6538.

### 5.2 Measurement results of the taxi travel demand

#### 5.2.1 Results of the temporal distribution of taxi travel demand

**Fig 9:**
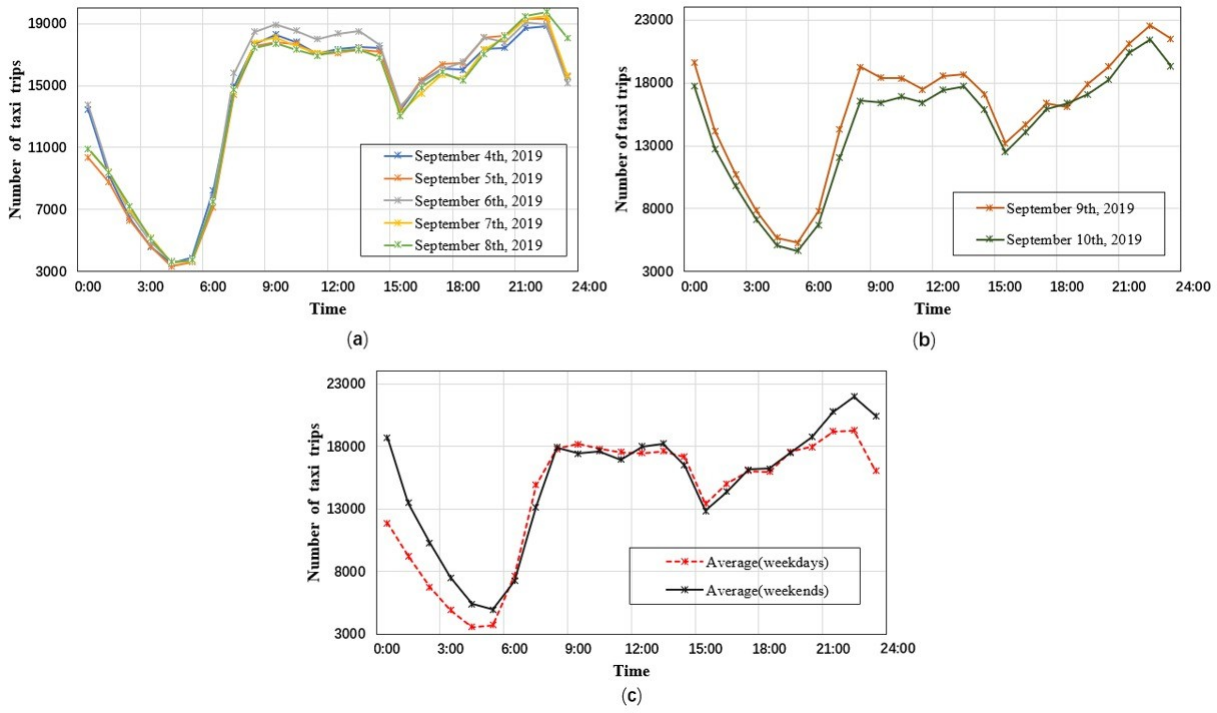
Taxi demand distribution in the main urban area of Xi’an. (a) Taxi demand average hourly from Monday to Friday; (b) taxi demand average hourly from Saturday to Sunday; (c) taxi demand per hour and per day.

Taxi operation is flexible, and is not only influenced by the arrangement of taxi dispatching companies, but also by the subjective judgment of taxi drivers. We used the extracted and preprocessed taxi track data in the main urban area of Xi’an from September 4th to September 10th, 2019, to calculate the average hourly taxi demand in the same time period from Monday to Sunday, record the change of taxi travel demand in one day. And averaged it according to a single working day and a single rest day to get the average hourly taxi demand on weekdays and weekends (Fig 9).

It can be seen from Fig 9 that the distribution of taxi travel demand at each time of the week was consistent with the travel rules of residents. There were three obvious peak periods: 8:30–10:30, 12:30–14:30, and 21:30–23:30. And the taxi travel demand was relatively high in the evening, and reached its maximum at about 22.30.

On weekdays, since the data extracted in this paper related to taxi drop-off points, there was a certain lag in time. Thus, the two peak periods, from 8:30 to 10:30 and from 12:30 to 14:30, corresponded to the commuting time of residents on weekdays. However, in the evening, some residents change from commuters to recreational travelers, and some buses in Xi’an stop operating after 19:30 or 20:30. Thus, a considerable portion of urban traffic demand is forced to turn to taxis and other means of transportation, increasing the number of residents’ taxi trips. As a result, the late peak period is pushed back from 17:30–19:30 to 21:30–23:30.

Compared with weekdays, during the periods of 0:00–6:00, 12:00–14:00 and 17:30–24:00 on weekends, the taxi travel demand was higher than that of working days, while other periods were lower than working days. This result is in line with residents’ daily travel habits. One reason is that most residents choose to rest on weekend mornings, they postpone their time to go out. Second, many residents choose to have leisure and entertainment activities on weekends, which always last until late at night. At this time, due to the suspension of buses, the probability of choosing to take a taxi to return to the residence increases, leading to an increase in the taxi travel demand. In general, taxi drivers can generate considerable income by attracting passengers after 21:00.

### 5.2.2 Results of the spatial distribution of taxi travel demand

**Fig 10:**
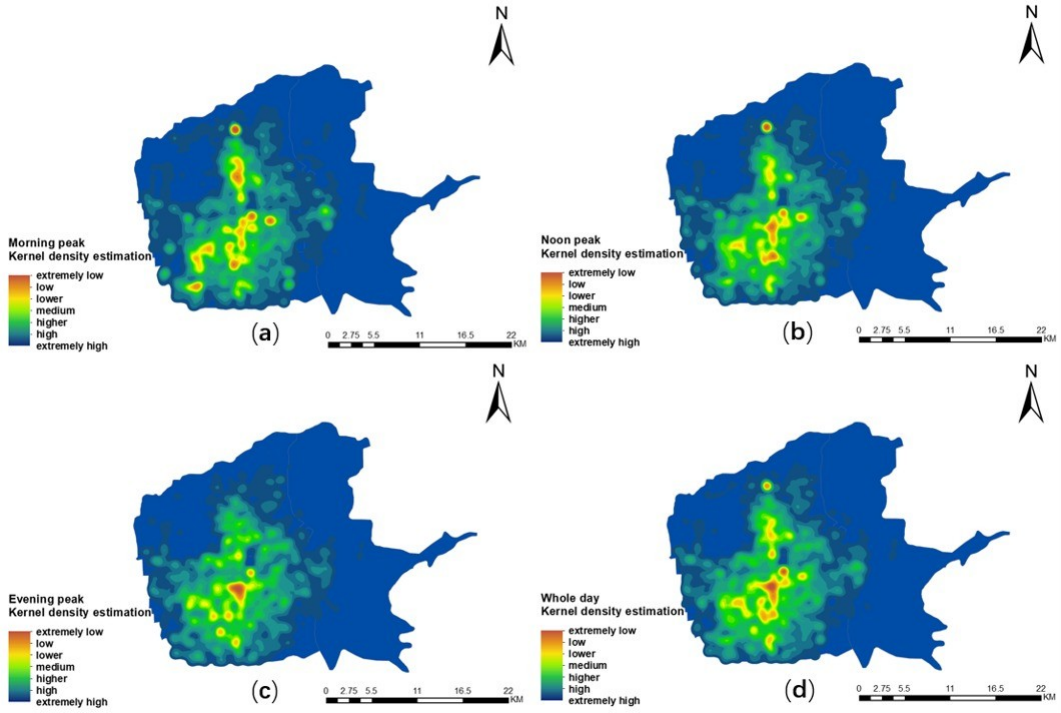
Spatial and temporal distribution characteristics of taxi travel demand on a single working day in the main urban area of Xi’an city. (a) Morning peak; (b) noon peak; (c) evening peak; (d) overall peak.

**Fig 11:**
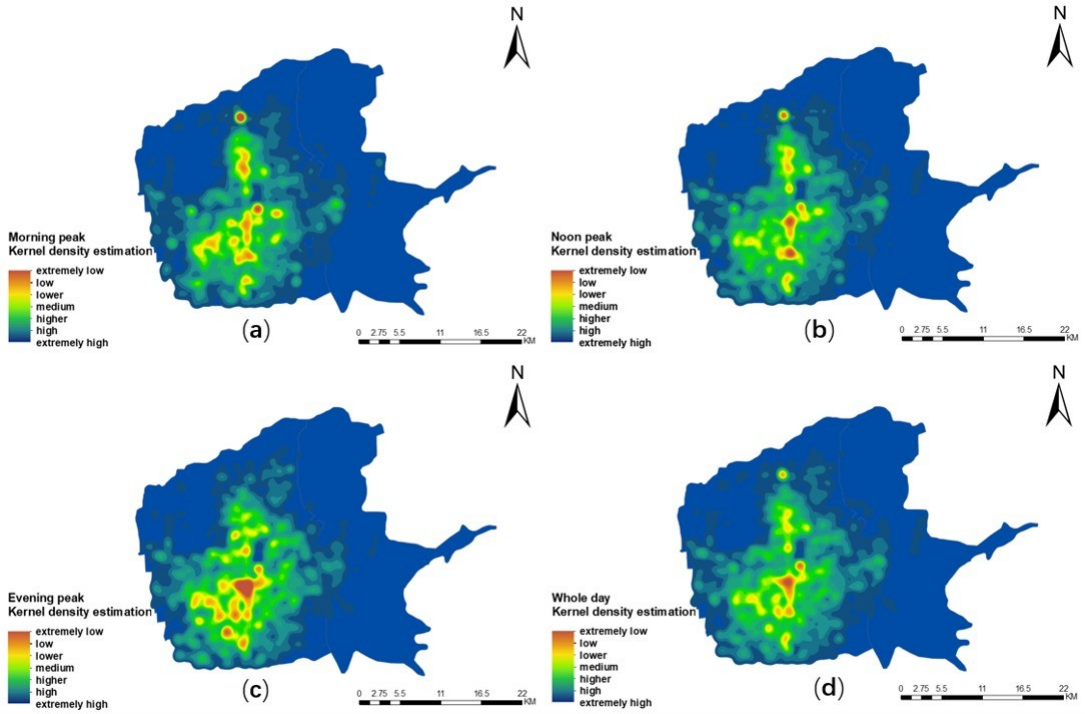
Spatial and temporal distribution characteristics of taxi travel demand on a single day off in the main urban area of Xi’an. (a) Morning peak; (b) noon peak; (c) evening peak; (d) overall peak.

In order to further study the agglomeration of taxi travel demand and its changes during different periods, this paper calculated the average hourly taxi demand of all grid cells in the same time period from Monday to Sunday. The calculation was based on the three time periods (8:30–10:30, 12:30–14:30, and 21:30–23:30) from Fig 9 to represent the morning, noon, and evening peaks of traffic in the main urban area of Xi’an respectively. The kernel density estimation method was used to analyze the time-space distribution and evolution characteristics of taxi travel demand during the three peak periods (Figs 10 and 11). It was found that different time spans presented obviously different travel patterns.

Figs 10D and 11D reveal the hot spot distribution of taxi travel demand in the main urban area of Xi’an for a whole day on weekdays and a whole day on weekends. It is found that the hot spots are similar, and they are mainly concentrated around the commercial centers of Yanta, Beilin, Xincheng, and Weiyang districts, as well as the High-Tech district with the good investment environment and active economic development. There are various types of functional facilities, such as catering, shopping, leisure and entertainment, life services, and offices. In addition, there are also hot spots around High-Speed and conventional Railway stations. This may be because the stations are surrounded by a large number of hotels, catering services, parking areas, etc. Outside of these areas, the taxi travel demand in other places is relatively weak. This indicates the spatial distribution of hot spots is related to the mixed degree of urban functions. That is to say, the taxi travel demand is affected by the mixed degree of urban functions, and the influence may be different in different spaces.

For the three peak periods on weekdays (Fig 10A–10C), it is found that the spatial distribution of the hot spots of residents’ taxi travel demand is constantly evolving and variable. Under the influence of the urban internal functions, Fig 10A represents that the hot spots are spread around the the Bell Tower, Xiaozhai, the High-Tech Industrial Park, the vertical section from Fengcheng No.1 Road to the City Sports Park, the High-Speed Railway Station, and the Xi’an Railway Station in the morning peak period. Fig 10B displays the noon peak period. The distribution of hot spots in the vertical section from Fengcheng No.1 Road to the City Sports Park has been reduced. This may be relevant to the midday rest system implemented by most enterprises. However, the distribution of hot spots near the Bell Tower, Xiaozhai, the Xi’an Railway Station, and the Exhibition Center has increased. Fig 10C represents that hot spots are more concentrated in the vicinity of the Bell Tower, the Electronics City, and the Exhibition Center during the evening peak period. Nevertheless, the taxi travel demand in the vertical section from Fengcheng No.1 Road to the City Sports Park is greatly reduced.

For the three peak periods on weekends (Fig 11A–11C), it is found that the hot spots are greatly reduced near the High-Tech Industrial Park in the morning peak period (Fig 11A). It may be that the High-Tech Industrial Park is the place for a large number of talents to work here, so the weekend off has caused a decrease in taxi travel. Fig 11B displays the noon peak period. The distribution of hot spots in the Bell Tower, Xiaozhai, and the Exhibition Center has been increased. This is connected to their irreplaceable geographical location, commercial status, etc. In Fig 11C during the evening peak period, multiple hotspots are formed. And the concentration range of the hot spots in the Bell Tower is greatly increased. This may be because residents often choose to go out for entertainment and shopping on weekend nights. However, the distribution of hot spots in the vertical section from Fengcheng No.1 Road to the City Sports Park is greatly reduced. On the one hand, this is related to the fact that it is the only route for residents in the northern suburbs to travel and the important transfer locations. On the other hand, it is related to the fact that some buses stop running after 19:30 and that consumers want to relax at night and are willing to pay higher transportation costs in exchange for more comfortable transportation services. They often choose to take a taxi directly from the starting point to the destination instead of taking a taxi to these exchange points for transfer.

To summarize, the above analysis indicates the influence of the mixed degree of urban functions on residents’ taxi travel demand in a city is not only different spatially, but also temporally. Thus, it is important to further clarify these differences.

### 5.3 Measurement results of geographically weighted regression

We further study the spatial differences of the influence of the mixed degree of urban functions on residents’ taxi travel demand, and the evolution in different peak traffic periods. This paper took each grid cell in the research area as the research unit, the taxi travel demand of residents in each grid cell as the dependent variable, and the mixed degree of urban internal functions in each grid cell as the explanatory variable to construct the global OLS and the GWR models on weekdays and weekends. The comparison results are as follows (Tables 4 and 5).

**Table 4:** Comparison results of the OLS and the GWR model on weekdays.

Diagnostics	Morning peak		Noon peak		Evening peak		Whole day	
	OLS	GWR	OLS	GWR	OLS	GWR	OLS	GWR
*R*^2^	0.1399	0.4308	0.1646	0.4895	0.1935	0.5454	0.1936	0.5422
$\overset{¯}{R^{2}}$	0.1397	0.3920	0.1643	0.4548	0.1933	0.5145	0.1934	0.5111
*AIC*_*C*_	45387	44264	44741	43340	44136	42453	57131	55474
Bandwidth	—	1883	—	1883	—	1883	—	1883

**Table 5:** Comparison results of the OLS and the GWR model on weekends.

Diagnostics	Morning peak		Noon peak		Evening peak		Whole day	
	OLS	GWR	OLS	GWR	OLS	GWR	OLS	GWR
*R*^2^	0.1437	0.4468	0.1648	0.4751	0.1786	0.5340	0.1899	0.5354
****$\overset{¯}{R^{2}}$****	0.1435	0.4091	0.1645	0.4394	0.1784	0.5023	0.1897	0.5037
*AIC*_*C*_	34258	33052	33817	32513	34942	33282	51242	49620
Bandwidth	—	1883	—	1883	—	1883	—	1883

It can be seen from Tables 4 and 5 that the adjusted *R*^2^ values of the GWR model are higher than those of the global OLS model whether on weekdays or weekends. These results all indicate that the GWR model considering temporal and spatial effects can improve the goodness of fit of the model obviously. Moreover, on weekdays, the *AIC*_*C*_ values of the GWR model are lower than those of the OLS model; the difference values are greater than 3. So it is also considered that the model with the lower *AIC*_*C*_ is better [44]. The results obtained at the weekend are the same. This again reveals that the GWR model is better than the OLS model in studying the impact of the mixed degree of urban functions on residents’ taxi travel demand. Further analysis of results is as follows.

**Fig 12:**
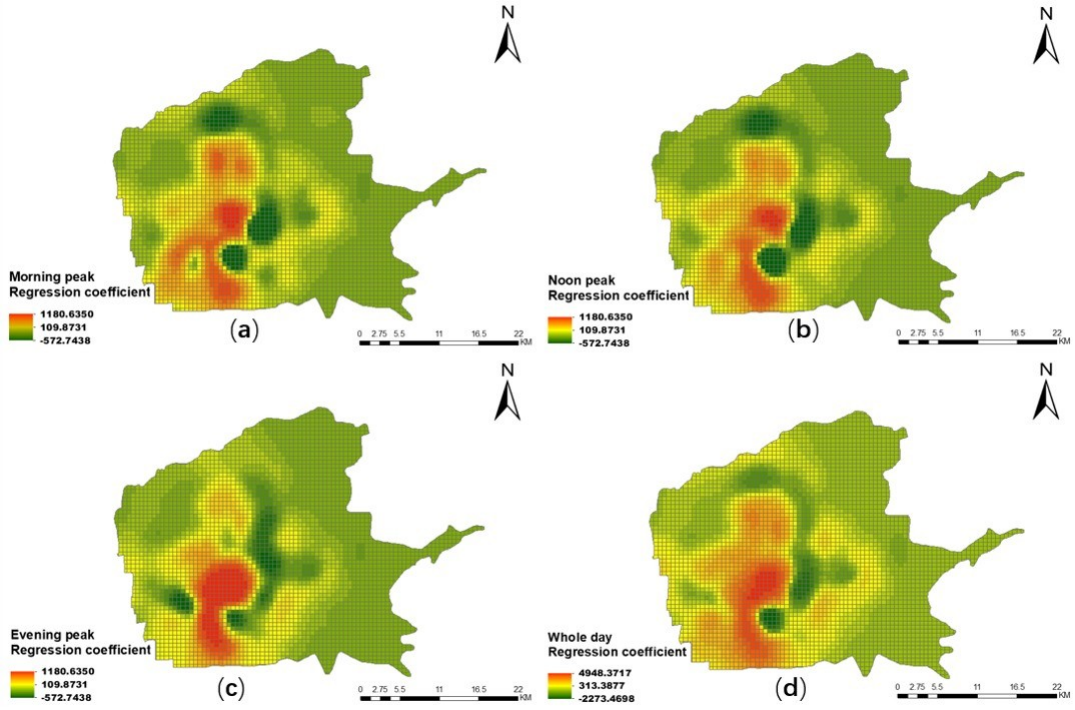
Spatial distribution of coefficient of the mixed degree of urban functions in different periods on weekdays. (a) Morning peak; (b) noon peak; (c) evening peak; (d) overall peak.

**Fig 13:**
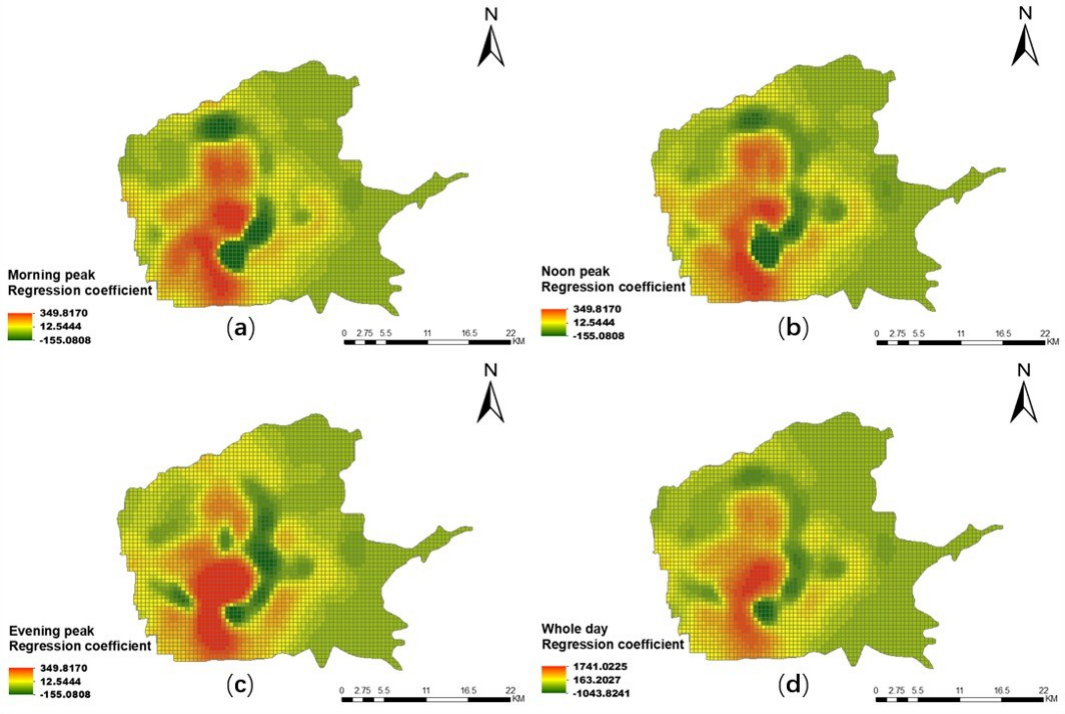
Spatial distribution of coefficient of the mixed degree of urban functions in different periods on weekends. (a) Morning peak; (b) noon peak; (c) evening peak; (d) whole day.

Figs 12 and 13 give the spatial distribution results of the *H*_*s*_ coefficient of the mixed degree of urban functions in the three peak periods and the whole day on weekdays and weekends, using the GWR Model. Generally, the impact of the mixed degree of urban functions on the residents’ taxi travel demand shows temporal and spatial differences.

From the perspective of spatial differences, whether on weekdays or weekends, compared with the north–south direction, the east–west direction has a greater difference in the impact of the mixed degree of urban functions on residents’ taxi travel demand. And in these two directions, the differences in the *H*_*s*_ coefficient on weekdays are greater than those on weekends. In addition, the regression coefficient values in the north-south direction are basically positive proportional, excluding the area near the High-Speed Rail Station and Xiaozhai. And the impact during the weekdays is greater than the weekends. Near the Electronics City, Xiaozhai, the Exhibition Center, and the High-Tech Industrial Park, the mixed degree of urban function has the greatest impact on residents’ taxi travel demand, and they are all in positive proportional. It shows that the higher the mixed degree of urban functions, the greater the taxi travel demand [45]. To a certain extent, this confirms that regions with high POI diversity are more attractive than other regions, which is consistent with Crane et al. [46], who believes that improving the accessibility of multiple destinations can increase travel demands. In particular, in the north-east, south-east and north-west corner of the main urban area, the mixed degree of urban functions has the little impact on the residents’ taxi travel demand, with most areas showing an inverse proportion.

From the perspective of temporal differences, the Bell tower, the Exhibition Center, the High-Tech Industrial Park, and the Xi’an Railway Station, as an important commercial center, high-tech area, and transportation hub, are irreplaceable in the city. They attract residents from other regions all day long. Therefore, in the three peak periods and the whole day on weekdays and weekends, the demand for taxi travel shows an increasing trend with the increase of the mixed degree of urban functions. However, compared with the morning and noon peak periods, the mixed degree of urban functions has less influence on the residents’ taxi travel demand in the evening peak period from the vertical section from Fengcheng No.1 Road to the City Sports Park. And the impact is less on weekdays than on weekends. In addition, whether on weekdays or weekends, near the High-Tech Industrial Park, the taxi travel demand decreases with the increase of the mixed degree of urban functions during the morning and noon peaks compared with the evening peak period. This may be caused by that the High-Tech Industrial Park is mainly based on a large number of work units, and most of the residents go home from work on weekdays or rest at home on weekends during the evening peak periods. And the increased mixing of urban POIs does not have a noticeable effect.

### 5.4 Measurement results of single urban function and mixed urban functions

Furthermore, in order to verify that the residents’ taxi travel demand is better affected by the interaction of urban internal functions than by the single urban function, this paper takes tourist attraction POI as an example to demonstrate. Based on the previous 13 classifications of urban POIs, we compared the results of the GWR model constructed by the urban POIs (13) and taxi travel demand, as well as single tourist attraction POI and taxi travel demand (Tables 6 and 7).

**Table 6:** Comparison results of the GWR model between urban POIs (13) and single tourist attraction POI on weekdays.

Diagnostics	Morning peak		Noon peak		Evening peak		Whole day	
	Urban POIs (13)	single tourist attraction POI	urban POIs (13)	single tourist attraction POI	Urban POIs (13)	single tourist attraction POI	urban POIs (13)	single tourist attraction POI
*R*^2^	0.4308	0.3425	0.4895	0.4091	0.5454	0.4680	0.5422	0.4636
$\overset{¯}{R^{2}}$	0.3920	0.3290	0.4548	0.3971	0.5145	0.4571	0.5111	0.4526
*AIC*_*C*_	44264	44546	43340	43629	42453	42781	55474	55806
Bandwidth	1883	3546	1883	3546	1883	3546	1883	3546

**Table 7:** Comparison results of the GWR model between urban POIs (13) and single tourist attraction POI on weekends.

Diagnostics	Morning peak		Noon peak		Evening peak		Whole day	
	Urban POIs (13)	single tourist attraction POI	urban POIs (13)	single tourist attraction POI	urban POIs (13)	single tourist attraction POI	urban POIs (13)	single tourist attraction POI
*R*^2^	0.4468	0.3601	0.4751	0.3974	0.5340	0.4530	0.5354	0.4574
$\overset{¯}{R^{2}}$	0.4091	0.3470	0.4394	0.3851	0.5023	0.4418	0.5037	0.4463
*AIC*_*C*_	33052	33338	32513	32774	33282	33621	49620	49940
Bandwidth	1883	3546	1883	3546	1883	3546	1883	3546

It can be seen from Tables 6 and 7 that on weekdays and weekends, regardless of the three peak periods or the whole day, the adjustment *R*^2^ values of the GWR model of urban POIs (13) are higher than those of the GWR model of single tourist attraction POI. And the *AIC*_*C*_ values of the GWR model of urban POIs (13) are lower than those of the GWR model of single tourist attraction POI. It shows that the GWR model of urban POIs (13) has higher goodness of fit. Despite this, we further removed the single tourist attraction POI and constructed the GWR model based on the remaining urban POIs (12) and taxi travel demand. The results are shown in Table 8.

**Table 8:** Results of the GWR model of urban POIs (12) on weekdays and weekends.

Diagnostics	weekdays				weekends			
	Morning peak	Noon peak	Evening peak	Whole day	Morning peak	Noon peak	Evening peak	Whole day
*R*^2^	0.3991	0.4608	0.5190	0.5155	0.4164	0.4468	0.5065	0.5090
$\overset{¯}{R^{2}}$	0.3717	0.4363	0.4971	0.4934	0.3899	0.4217	0.4840	0.4867
*AIC*_*C*_	44349	43426	42546	55568	33133	32592	33378	49708
Bandwidth	2307	2307	2307	2307	2307	2307	2307	2307

Comparing the results of Tables 6–8, it is found that on weekdays and weekends, regardless of the three peak periods or the whole day, the adjustment *R*^2^ values of the GWR model of urban POIs (13) are highest, followed by urban POIs (12), and single tourist attraction POI. The *AIC*_*C*_ values of the GWR model of urban POIs (13) are lowest. And the *AIC*_*C*_ values of the GWR model of single tourist attraction POI have the highest *AIC*_*C*_ values. It indicates that the GWR model of urban POIs (13) is the most effective, followed by POIs (12), and single tourist attraction POI.

To sum up, the taxi travel demand, that is, the purpose of travel, which is largely influenced by the interaction of urban internal functions. Although the single function of the city has an impact on the taxi travel demand, the result of the single function is not ideal. It fully explains the importance of the connection between the multifunctional living environment and residents’ taxi travel demand.

## 6. Conclusions

With the rapid development of urbanization, the continuous growth of the income level of urban residents, and the popularity of the pursuit of a high-quality, convenient life and consumption, the demand for taxi travel continues to have great potential. Due to limited taxi resources and the uncertainty of residents’ daily activities, it is an urgent issue for governments to make relevant policies and measures to provide support for residents’ taxi travel. It is necessary to further explore the distribution of residents’ travel purposes, namely, the POIs of basic urban constituent units, and the relationship between POIs and residents’ travel needs. Therefore, this paper calculates and analyzes the mixed degree of functions within a city based on urban POI data. The kernel density estimation method is used to visualize the distribution of taxi travel demand. And the GWR model is applied to identify the spatial variation of the coefficient of the influence of the mixed degree of urban functions on the residents’ taxi travel demand in the main urban area of Xi’an. It also compares the effects of the single function and the mixed functions on taxi travel demand of the city. The results of the discussion and analysis can be summarized as follows.

First, in the main urban area of Xi’an City, the overall pattern of the mixed degree of urban functions is lowest outside the third ring road, and gradually increasing within the third ring road. Understanding these distribution characteristics can provide a reference for residents’ diversified travel purposes.

Second, in the main urban area of Xi’an, the taxi travel demand on weekends is higher than that on weekdays during the periods of 0:00–6:00, 12:00–14:00, and 17:30–24:00. And there is a high taxi travel demand during the evening peak (21:30–23:30) on weekdays and weekends. The management department can take these periods into account when carrying out dynamic adjustment of transport capacity, and formulate taxi capacity delivery measures in different periods.

Third, in the main urban area of Xi’an City, there are several hot spots with stable taxi demand during the morning, noon, and evening peak periods, that is, there is a fixed population but also some differences in time. For example, during the noon peak (12:30–14:30) and evening peak (21:30–23:30), the taxi travel demand around the Bell Tower, Xiaozhai, and the Exhibition Center is stable. For these hot spots of taxi travel demand, the taxi management department can allocate taxis reasonably. And encourage residents to stagger the peak travel periods when going to these places, so as to ensure the convenience and smooth travel of residents.

Fourth, in the main urban area of Xi’an, the east-west direction of Xi’an reveals a great difference in the influence of the mixed degree urban functions on the residents’ taxi travel demand compared with the north and south sides. In the north-east, south-east and north-west corner of the main urban area, the mixed degree of urban functions has the least impact on the residents’ taxi travel demand, and basically in inverse proportion. Near the Bell Tower, Xiaozhai, the Exhibition Center, and the High-Tech Industrial Park, where there is a high mixed degree of urban functions, and the residents’ taxi travel demand is the largest. With this information, the management department can consider the dynamic car allocation algorithm affected by the mixed degree of urban function when the taxi capacity is configured. Taxi drivers can also take into account the differences in temporal and spatial to attract passengers, so as to alleviate the traffic congestion and improve the operating efficiency of taxi drivers.

Fifth, the taxi travel demand is largely influenced by the interaction of the urban internal functions. Although the single function of the city has an impact on taxi travel demand, the result of the single function is not ideal. Therefore, in the process of urban planning, the optimal combinations of the basic units of urban space should be further explored.

## 7. Limitations and future work

Further research should address the deficiencies of this paper, which are as follows:

1. The classification of urban POI data types in this paper is partly based on the needs of this study, and more research on the classification of urban POI data needs to be carried out.
2. This paper takes the big city Xi’an as an example to research, which is different from small and medium-sized cities. In the future, we can carry out research on small and medium-sized cities to further verify our conclusions.
3. This study focuses on the impact of the mixed degree of urban functions on the taxi travel demand, without considering factors such as road capacity and traffic congestion. The influence of such factors can be further considered in the future.

## Supporting information

S1 File POI data. (RAR)

S2 File 11.4 taxi data. (RAR)

S3 File 11.5 taxi data. (RAR)

S4 File 11.6 taxi data. (RAR)

S5 File 11.7 taxi data. (RAR)

S6 File 11.8 taxi data. (RAR)

S7 File 11.9 taxi data. (RAR)

S8 File 11.10 taxi data. (RAR)
